# Spinal Cord Glioneuronal Tumor with Rosetted Neuropil-Like Islands in Pediatric Age Group

**DOI:** 10.1155/2014/471645

**Published:** 2014-12-10

**Authors:** Nil Comunoglu, Ozgur Kilickesmez, Buge Oz

**Affiliations:** ^1^Department of Pathology, Cerrahpasa Faculty of Medicine, Istanbul University, 34000 Istanbul, Turkey; ^2^Department of Radiology, Faculty of Medicine, Yeditepe University, Istanbul, Turkey

## Abstract

Glioneuronal neoplasms are rare tumors. Recently, an unusual glioneuronal tumor histologically showing neuropil-like islands has been described. Here, we present such a tumor originating from spinal cord of a 14-year-old girl, who has scoliosis and urinary incontinence. Microscopically, the glial component was chiefly fibrillary astrocytic, punctuated by neuropil-like islands. Immunohistochemically, glial tissue was GFAP positive, and neuropil-like areas and big neurons were synaptophysin reactive. For astrocytic component Ki-67 proliferation index was 1% and p53 was immunonegative. This case is unique in that in the literature it is the second reported case in pediatric age group that is located at spinal cord.

## 1. Introduction

Three new entities have been recently added to the group of glioneuronal tumors by the recently updated World Health Organization (WHO) Classification of Tumors of Central Nervous System [1]. Evolution of this classification has been achieved by recent positive results of immunohistochemical and molecular studies. The new entities are papillary glioneuronal tumor (PGT), rosetted glioneuronal tumor with neuropil-like islands, and rosette-forming glioneuronal tumor (RGNT) of fourth ventricle. These tumors, morphologically resembling glial neoplasms, have readily been identified by neuronal differentiation [1–3]. In particular, rosetted glioneuronal tumor with neuropil-like islands should be distinguished from RGNT and PGT. The lesion currently is considered as a variant of astrocytomas, WHO Grade II or Grade III [1]. In 1999, Teo et al. reported a series of 4 cases of a glioneuronal tumor of the adult cerebrum that were marked by neuropil-like or rosetted islands, otherwise resembling diffusely infiltrating astrocytomas [4]. Most cases in the literature have been located in the cerebrum [2–8]. Spinal cord localization has been reported very rarely [9–11]. The present case is the second rosetted glioneuronal tumor with neuropil-like islands occurring in childhood period localized at spinal cord.

## 2. Case Report

### 2.1. Clinical Summary

**Figure 1:**
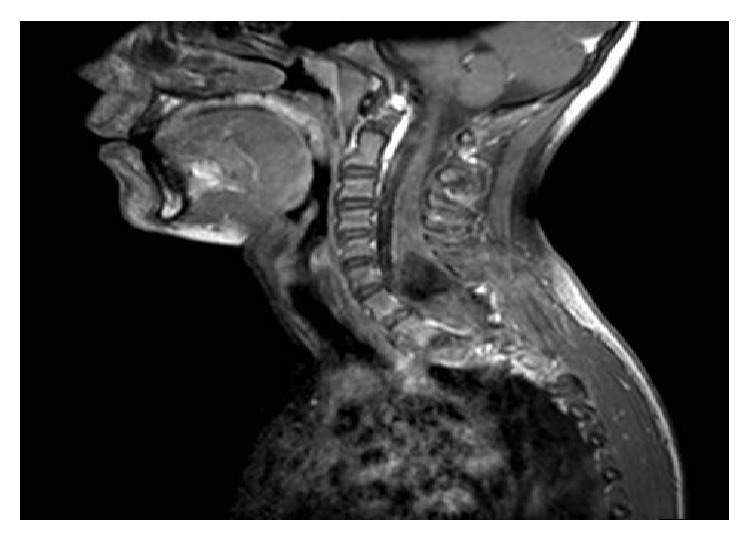
Sagittal fat saturated postcontrast spin-echo (SE) T1-weighted MRI of the cervical spine demonstrates a large expansile mixed solid and cystic intra-axial mass of the spinal cord extending from C5 to T1 level. Both the upper cystic and the lower solid components do not enhance.

**Figure 2:**
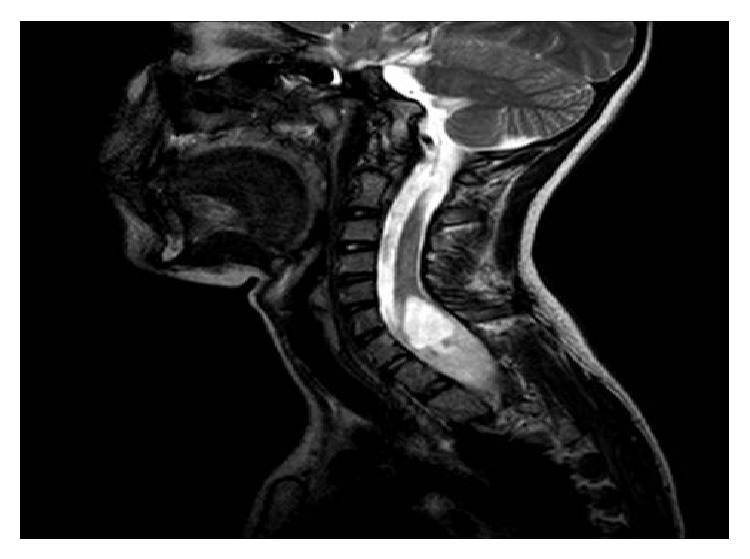
Sagittal turbo spin-echo (TSE) T2-weighted MRI of cervical spine demonstrates a large expansile intra-axial mass of the spinal cord. The upper cystic part and the lower solid parts of the mass are hyperintense.

We present a 14-year-old female patient followed up because of her scoliosis and urinary incontinence. Cervical and thoracic magnetic resonance imaging (MRI), performed during follow-up of her scoliosis, revealed a cervical intrathoracic mass lesion. The patient was then referred to the neurosurgery department. MRI revealed that an intramedullary mass lesion containing solid and cystic components was detected located at C5–T5, resulting in cervical scoliosis with right concave side and an expansion in spinal canal. Solid component of the mass was extending through C7–T3 vertebrae and was measuring 51 × 18 × 22 mm. This solid component of the tumor was heterogeneously hypointense on T1 image and heterogeneously hyperintense on T2 image (Figures 1 and 2). Surgically, C5, 6, 7 and T1 hemilaminotomy and intradural intramedullary tumor excision were performed.

### 2.2. Pathological Findings

**Figure 3:**
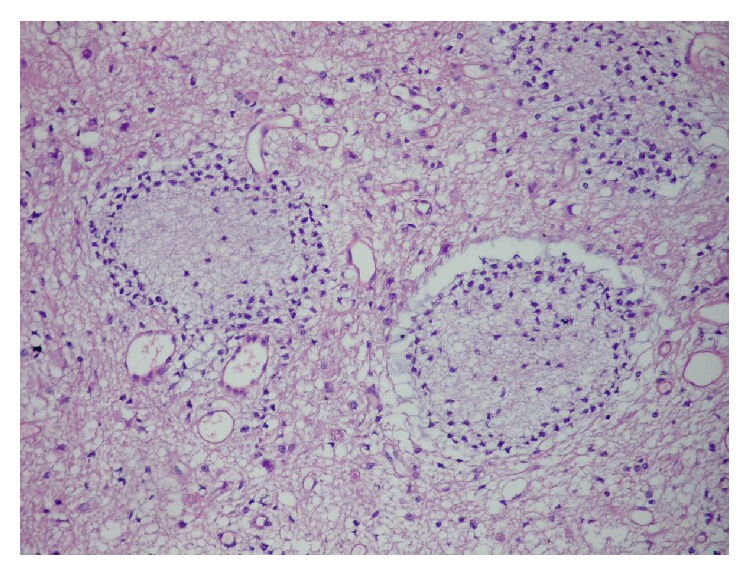
Oligodendrocyte-like cells and neuropil-like islands (H&E; ×200).

**Figure 4:**
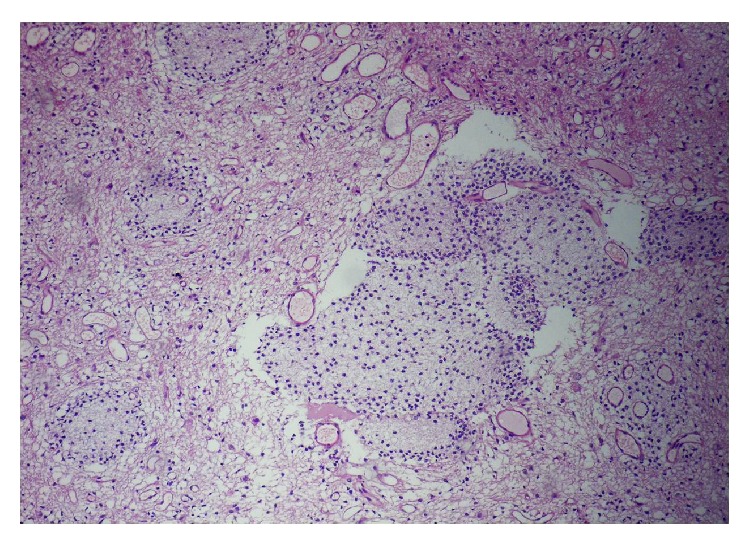
Neuropil-like island (H&E; ×100).

**Figure 5:**
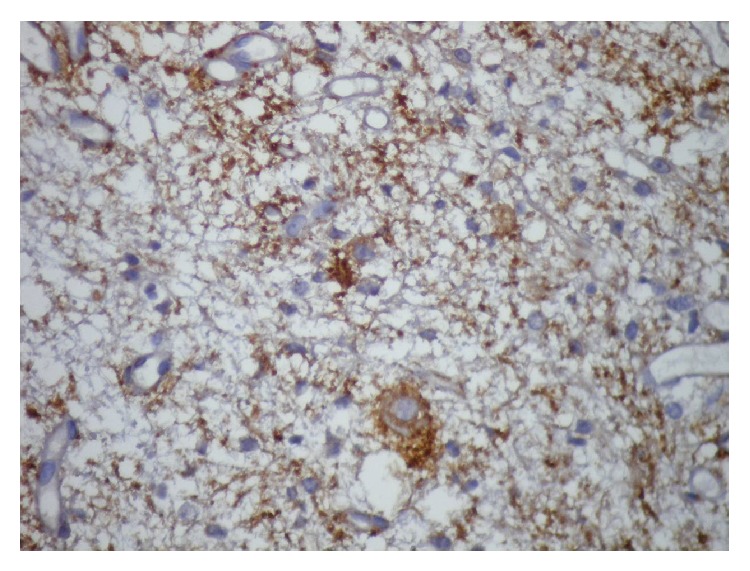
Synaptophysin positivity in large neurons (synaptophysin; ×400).

To the pathology department, yellowish 11 irregular tissue fragments, with the greatest measuring 6 × 4 × 2 mm, were submitted. Microscopic examination showed a mixed glioneuronal tumor composed of oligodendrocyte-like cells constituting neuropil-like islands, astrocytic cells, and neurons scattered in glial tissue (Figures 3 and 4). Astrocytic component consisted of microcystic fibrillary regions which does not contain Rosenthal fibers. Astrocytic component of the tumor did not contain mitosis, vascular proliferation, or necrosis. The tumor, graded according to the astrocytic component, was considered as WHO Grade 2. Immunohistochemically, glial tissue was GFAP positive (clone 6F2, DAKO), and neuropil-like areas and big neurons were synaptophysin positive (clone SY38, DAKO) (Figure 5). In astrocytic component Ki-67 (clone MIB-1, DAKO) proliferation index was 1%. p53 (clone 318-6-11, DAKO) was immunonegative. Consequently, the final diagnosis was spinal cord glioneuronal tumor with rosetted neuropil-like islands—WHO Grade II.

## 3. Discussion

Our case is a childhood glioneuronal tumor with rosetted neuropil-like islands located at the cervicothoracic region of the spinal cord. This group of tumors was firstly described by Teo et al. [4]. These tumors usually present with supratentorial, hemispheric location. Reported patients are between the ages of 23 and 44 [2–8]. Harris and Horoupian, Rickert et al., and Poliani et al. have reported cases of spinal cord location [9–11]. To the best of our knowledge, our case is the second youngest case having this tumor at the spinal cord.

Radiologically, intramedullary tumors are usually described as nodular, moderately contrast enhancing lesions. Cerebral cases are hypointense in T1 and hyperintense and focally contrast enhancing in T2. Hemispheric white matter, corpus callosum, and basal ganglia locations have been reported [2–5, 9].

These tumors are morphologically biphasic tumors composed of astrocytic and neuronal components. Distinct from diffuse astrocytomas, neuropil-like islands disperse in neuroparenchyma in permeative fashion. In some cases, astrocytic component may be dominant. Microcystic and myxoid changes can be detected within these regions. Cellular component of neuropil-like islands are oligodendrocyte-like cells displaying perinuclear clearing. Big neurons are scattered in glial tissue. They do not show pleomorphism or binucleation [2–5].

Immunohistochemically, glial tissue is positive for GFAP; neuropil-like islands and big neurons are positive for neuronal markers such as synaptophysin, Neu-N, and Hu [1]. Keyvani et al. reported that, within these tumors, Ki-67 proliferation index was 3.9% (0.6–9.3) in neuronal component and 1.5% (0.6–2.6) in astrocytic component [3]. In our case, Ki-67 proliferation index was 1% in astrocytic component and it was negative in neuronal component. Positivity for p53 has been reported in some cases [5]. p53 was negative in our case.

The glioneuronal tumors can be mistakenly diagnosed as oligodendrogliomas, astrocytomas, or ependymomas. Oligodendrogliomas are distinct with their molecular genetic features. Rarely, ependymomas containing neuropil-like islands have been reported [1]. Ependymomas can be distinguished morphologically by displaying formation of rosettes and pseudorosettes and immunohistochemically by EMA positivity. Perry et al. claimed that glioneuronal tumors were oligodendroglial tumors showing neurocytic differentiation [12]. Oligodendrocyte-like cells having perinuclear halo and 1p 19q deletion in a small number of cases are histologic and molecular genetic findings, respectively, supporting Perry and his colleagues' claim [12, 13]. However, it has been noted that in some cases 1p 19q deletion could not be detected [8]. On the contrary, Keyvani et al. found increase in 7q and loss in 9p in molecular genetic studies of a small number of cases [3]. Loss of 4q, 5q, and 11p and gain of 6p, 7q, 8q, 11q, 12p, and 15q were found in another cytogenetic study of Min et al. [14].

Actually these tumors can be diagnosed morphologically straightforwardly. They are classified within the group of astrocytomas; however, their category can be changed in the future classifications. Their rarity results in classification difficulties. It would be beneficial to collect cases in a center in order to conduct molecular studies and report the findings.
